# Dietary specialization is linked to reduced species durations in North American fossil canids

**DOI:** 10.1098/rsos.171861

**Published:** 2018-04-25

**Authors:** Mairin Balisi, Corinna Casey, Blaire Van Valkenburgh

**Affiliations:** 1 Department of Ecology and Evolutionary Biology, University of California, Los Angeles, CA, USA; 2 Vertebrate Paleontology Department, Natural History Museum of Los Angeles County, Los Angeles, CA, USA

**Keywords:** carnivory, functional traits, macroevolution, macroecology, multivariate analyses, phylogenetic comparative methods

## Abstract

How traits influence species persistence is a fundamental question in ecology, evolution and palaeontology. We test the relationship between dietary traits and both species duration and locality coverage over 40 million years in North American canids, a clade with considerable ecomorphological disparity and a dense fossil record. Because ecomorphological generalization—broad resource use—may enable species to withstand disturbance, we predicted that canids of average size and mesocarnivory would exhibit longer durations and wider distributions than specialized larger or smaller species. Second, because locality coverage might reflect dispersal ability and/or survivability in a range of habitats, we predicted that high coverage would correspond with longer durations. We find a nonlinear relationship between species duration and degree of carnivory: species at either end of the carnivory spectrum tend to have shorter durations than mesocarnivores. Locality coverage shows no relationship with size, diet or duration. To test whether generalization (medium size, mesocarnivory) corresponds to an adaptive optimum, we fit trait evolution models to previously generated canid phylogenies. Our analyses identify no single optimum in size or diet. Instead, the primary model of size evolution is a classic Cope's Rule increase over time, while dietary evolution does not conform to a single model.

## 1. Background

The potential to predict emergent species- and community-level patterns and processes from functional traits is of great ecological and evolutionary interest [1–3]. For example, which traits cause some species to be more widespread and last longer than others? Using geographical breadth and species longevity as measures of success, how do a species' traits interact to make it successful over evolutionary time?

In extant mammals, abundance, range size and population size are measures of success that can be influenced by a variety of factors, such as body size [4,5], diet or prey choice [6], and dispersal ability [7]. Fossil ecosystems provide another dimension—time—and permit the analysis of taxon longevity in addition to geographical range as a metric of success. In this study, we examine the impact of body size and diet on patterns of success in the family Canidae of the order Carnivora, which spans over two orders of magnitude in mass [8] and varies widely in diet from hypocarnivores with diets comprising less than 50% meat, to mesocarnivores with diets comprising 50–70% meat, to hypercarnivores with diets comprising over 70% meat [9]. On an ecomorphological spectrum from generalization to specialization, small-bodied hypocarnivory and large-bodied bone-cracking hypercarnivory form opposite specialized extremes.

### 1.1. Ecomorphological specialization in body size

Body size exerts pervasive effects on a variety of traits, such as habitat selection and resource use. As these traits form a species' niche [10,11], they may ultimately influence interactions at various scales ranging from community species assembly to continental clade dynamics [12].

Because energetic requirements scale allometrically with body size, animals tend to specialize on prey sizes that maximize their net energy gain while foraging [13]. To sustain high metabolic rates, small carnivorans and other small mammals spend most of their time foraging, specializing on high-energy foods—such as insects—that are available in sufficient supply and accompanied by low costs [12,14]. Additionally, small carnivorans are biomechanically and morphologically limited to small prey [15]; mid-sized and larger carnivorans are better equipped to eat a wider range of prey sizes [16]. Meanwhile, large predators specialize on large prey because small prey are insufficient to sustain the energetic costs of large size [13,15,17]. Therefore, while mammals on the size extremes might approach morphological and physiological constraints, mid-sized mammals are well within these limits [18,19].

Additionally, mid-sized mammals tend to inhabit a wide range of habitats, while large and small species are distributed relatively narrowly [20]. This is probably because small size limits the dispersal of small species, while the energetic costs associated with large size [17] constrain large species to live in habitats with high productivity. Small mammals also exhibit greater turnover among habitats, suggesting that—because of energetic and physiological constraints—they are limited by environmental variation or biotic effects more than are mid-sized and large mammals [12]. In these ways, medium size might be considered a generalist strategy, and small and large body sizes as specialist strategies.

### 1.2. Ecomorphological specialization in diet

Many previous workers have examined dietary ecomorphological specialization in extant and extinct carnivorans [9,21–24], including not only hypercarnivory but also hypocarnivory [25–28]. While hypocarnivores tend to have a varied diet, hypocarnivory—like hypercarnivory—constitutes an ecomorphological specialization for carnivorans, the earliest of whom originated with a full complement of teeth (12 molars, 16 premolars, 4 canines, 12 incisors). This ancestral dentition was equipped with blades to slice meat and basins to grind plant matter, enabling early canids to be as omnivorous as raccoons today. This ecomorphologically generalized toolkit provided the foundation for carnivorans to diversify into a range of diets.

Over evolutionary time, carnivorans could deviate from this generalized mesocarnivorous morphology by modifying the toolkit, largely by tooth loss, to emphasize some functions over others. A few carnivoran lineages reduced their premolars but enlarged the grinding area, with some clades flattening and co-opting the blades as grinding surfaces, reducing their ability to process significant portions of meat: the hypocarnivorous specialization (e.g. bears). By losing posterior molars, other carnivoran lineages reduced grinding area and therefore their ability to process plant matter: the hypercarnivorous specialization (e.g. cats). A further specialization of hypercarnivory is bone-cracking, a behaviour that provides access to nutritious marrow [29] but also requires a robust skull and dentition (e.g. hyenas) [30,31].

Because lost structures rarely re-evolve (Dollo's Law) [32], the loss of dental cusps and of teeth themselves is often irreversible. Lineages that have begun to venture down the path of ecomorphological specialization tend to be able to modify only features that remain. Therefore, carnivoran lineages that modify the mesocarnivorous morph—whether in the hyper- or hypocarnivorous direction—tend to become progressively more specialized over evolutionary time (electronic supplementary material, figure S1). As a consequence, relative to their ancestors, ecomorphological specialists may have reduced evolvability, or capacity to generate heritable phenotypic variation [33,34], which narrows their response to selection in evolutionary time.

### 1.3. Canidae

The fossil record of the carnivoran family Canidae includes a diversity of species that span and perhaps surpass the size and dietary spectrum of extant caniforms. Fossil canids (dogs) arose in North America in the late Eocene (approx. 40 million years ago, Ma), radiating into over 130 species in three subfamilies [35–37]: the extant Caninae; and Hesperocyoninae and Borophaginae, both of which are extinct. Originating as small-bodied forms with omnivorous adaptations, Hesperocyoninae and Borophaginae evolved towards large size (greater than 90 kg) [38] and hypercarnivory prior to extinction, suggesting a macroevolutionary ratchet in which dietary specialization along with reduced population densities heightened extinction risk [23,39]. Preliminary work suggests that Caninae follows a similar trend over its recent history, raising the question of their current vulnerability to extinction.

Ecomorphological specialization is readily observable in the canid cranio-dentition. The most hypercarnivorous canids (e.g. *Enhydrocyon*, *Epicyon*) have drastically reduced or lost the grinding molars, losing a cusp on the grinding basin of the lower carnassial to co-opt the now-trenchant basin as an extension of the carnassial blade. Meanwhile, the most hypocarnivorous canids (e.g. *Cynarctoides*, *Cynarctus*) bear dentition quite odd for canids: instead of cusps that could hold or pierce vertebrate meat, their teeth bear ridges that converge on ungulate selenodont morphology: a specialization for chewing tough plant matter (electronic supplementary material, figure S2). The existence of large-bodied hypercarnivorous adaptations as well as less-studied hypocarnivorous specializations makes fossil Canidae an ideal system within which to explore the effects of ecomorphological specialization on taxon success.

### 1.4. Aims

Previous work [39] has suggested that specialization for hypercarnivory heightened extinction risk in Hesperocyoninae and Borophaginae. However, these authors did not look at the opposite end of the spectrum: hypocarnivores. Additionally, this study found a positive correlation between carnivory and body size, but only a negative qualitative association—no significant statistical correlation—between carnivory and species duration [39]. Larger canids tend to be more carnivorous, and more carnivorous canids appear to have shorter durations, but the signal is noisy. Here, we follow up on this work by (a) refining estimates of duration, (b) doubling the number of species analysed, (c) including the third canid subfamily, fossil Caninae, (d) examining hypocarnivores as well as hypercarnivores, (e) tracking a geographical metric of success, locality coverage and (f) analysing the data within a phylogenetic context.

We test the null hypothesis that (a) body size and (b) diet (carnivory) have no relationship with either (a) species duration, a measure of success in time, or (b) occupancy or fossil locality coverage, a measure of success in space. We expect that generalized species of average size and mesocarnivorous diet will survive longer and have broader distributions than more specialized species because their flexibility allows them to better survive times of disturbance and exist over a wider range of environmental conditions. Moreover, using locality coverage (a measure of geographical range) as a proxy for dispersal ability, we predict that the combined effects of size, diet and locality coverage will better describe patterns of duration than the individual and combined effects of size and diet alone. Lastly, if generalization were advantageous, then medium size and mesocarnivory would correspond to an adaptive optimum, and the best-supported evolutionary model for both body mass and carnivory would be a single-peak Ornstein–Uhlenbeck model, where species traits would be constrained around optimum values (table 1).

**Table 1.** Summary statistics and measures of phylogenetic signal for the two intrinsic traits and two emergent properties.

metric	median	median absolute deviation	Pagel's *λ*	Pagel's *λ p*	Blomberg's *K*	Blomberg's *K p*
log_10_ body mass	log_10_0.971 (9.354 kg)	log_10_0.383 (7.611 kg)	0.992	7.42 × 10^−39^	3.06	0.001
carnivory	0	0.8827668	0.891	4.89 × 10^−16^	0.947	0.001
duration	3.688 Ma	3.117 Ma	0.891	0.00291	0.34	0.012
maxLocCover	0.1483515	0.1272834	6.61 × 10^−5^	1	0.262	0.279

## 2. Methods

### 2.1. Calculating species traits

#### 2.1.1. Body size

We estimated fossil canid body masses from the length of the lower first molar (m1 L) using a previously published regression equation based on extant Canidae [40].

#### 2.1.2. Carnivory

We collected measurements of six commonly used characters [27,41,42] of the skull, jaw and dentition for a comparative dataset of 45 extant caniform and hyaenid species and a dataset of 131 fossil canid species. Following Van Valkenburgh *et al.* [39], we combined the species means of these characters into three ratios of dietary significance: RBL, relative blade length (trigonid blade length relative to total length of lower first molar); RUGA, relative upper grinding area (square root of upper molar grinding area relative to upper fourth premolar length); and JD/DL, jaw depth relative to dentary length. For species that we could not measure ourselves, we obtained character means or dietary indices from published work [35–37,39,43,44], permitting expansion of our analysis to a broad range of extant carnivorans and fossil canids. Because fossils are often fragmentary, recording all characters for all species was not possible; our largest sample size is 117 species for the characters RBL and m1 L. The extant taxa, coded dietary categories and references for dietary categories are in electronic supplementary material, table S1.

The dietary ratio JD/DL requires complete dentaries, which are often not available for fossil species. To maximize recovery of this ratio, we developed an equation relating the length of the lower first molar to dentary length for each canid subfamily and used this to estimate dentary length in species without complete dentaries (electronic supplementary material), a process that revealed subtle differences in the jaw morphologies of the three subfamilies. In comparison to hesperocyonines and canines, borophagines—including smaller, putatively non-bone-cracking members of the subfamily—tend to have shorter jaws relative to the length of the lower first molar (electronic supplementary material, figure S3).

We ran principal components analysis (PCA) on the three ratios for the 45 extant taxa (electronic supplementary material, table S1) using the R function prcomp(), and used the first principal component axis as a ‘carnivory index’. The variances of the ratios differ by an order of magnitude (median absolute deviations for extant-species ratios: RBL, 0.039; RUGA, 0.203; JD/DL, 0.027) and would be disproportionately weighted in a PCA using the covariance matrix. Because of this, we used the correlation matrix instead, which rescales and standardizes the data. The principal component analysis based on extant taxa of known diet generated a multivariate linear model, which we then used with the R function predict() to predict numerical values of the fossil taxa of unknown diet. These numerical predictions for extinct taxa were graphically superimposed onto the model of the extant taxa. In this visualization, extinct taxa are most similar in dietary ecomorphology to the closest-plotting extant taxa.

A discriminant function analysis would be appropriate if classifying taxa of unknown diet according to a comparative dataset of discrete categories; however, because extant dietary categories may not adequately describe fossil species' dietary adaptations, we did not classify fossil taxa to the extant categories. Therefore, identification of dietary categories for extant taxa was only for visualization. While not correcting for phylogeny during preliminary data reduction (e.g. standard PCA) can produce misleading results in subsequent phylogenetic comparative analysis [45], phylogenetic PCA requires assuming an evolutionary model to generate the principal component scores, which may also distort subsequent phylogenetic comparative analysis if the true model differs from the assumed model [46]. Therefore, we performed standard PCA followed by phylogenetic comparative methods.

### 2.2. Calculating success in space and time for fossil canids

We compiled occurrence data for North American fossil canids from the Neogene Mammal Mapping Portal (NeoMap, http://ucmp.berkeley.edu/neomap [47,48]) and Fossilworks/Paleobiology Database (http://www.fossilworks.org; http://www.paleobiodb.org). We last accessed the databases on 24 March 2017, cross-checking database records against the canid monographs by Wang *et al.* [35–37] and more recent occurrences in the literature [49–51]. In cases of overlap between the two databases, we used the occurrence record from NeoMap, because NeoMap's maximum and minimum age records, when cross-checked against the literature, were more precise than those of Fossilworks/the Paleobiology Database, which assigns dates based on the occurrence or locality's time interval and therefore tends to be of more variable precision.

We calculated two emergent properties for each species: (a) sampling-adjusted species duration, a measure of success in time, and (b) maximum occupancy or locality coverage, a measure of success in space. We excluded singletons (*n* = 23) or species that occur at only one locality, because calculations of duration and locality coverage for these would be disproportionately biased by poor preservation. We also excluded extant species (*n* = 8), because their geographical ranges probably have been constrained by anthropogenic activities and structures. Altogether, 107 non-singleton extinct species were included in the maximum dataset. These, the excluded singletons, and extant species totalled 3710 fossil occurrences ranging from 38.713 (±0.951) to 0.0 (±0.0) Ma.

#### 2.2.1. Species duration

Because the fossil record does not preserve all individuals that have ever existed, the first appearance date (FAD) is unlikely to capture the first individual after a given species originated, and the last appearance date (LAD) is unlikely to capture the last individual before a given species goes extinct. To account for this incomplete preservation, we calculated sampling-adjusted species durations using the open-source Python program PyRate [52] and a Markov chain Monte Carlo birth-death model run for the default setting of 10 000 000 iterations, with the first 200 000 discarded as burn-in. This process yielded skewed distributions of times of speciation (TS, right-skewed) and extinction (TE, left-skewed) for each species. We chose the medians of these distributions as unbiased estimates of TS and TE [5], and calculated the sampling-adjusted duration for each species as median(TE) minus median(TS).

#### 2.2.2. Maximum locality coverage (occupancy)

Similarly, because not all environments are conducive to fossilization, the fossil record probably captures only part of the geographical range that a species has occupied over its duration. Additionally, some fossil localities are clustered more closely than others, so that counting only the number of localities where a species is found would overestimate the range of species abundant at a given cluster even if the species is not actually widespread [6,53]. To account for preservational bias and clustering of localities, we divided the North American continent into standardized half-degree by half-degree ‘cells’ and calculated occupancy as a proportion of the number of cells occupied by a given species out of the number of cells occupied by all canid species for a given time period [54,55]. Calculating geographical occupancy as a proportion of available localities for a given time interval provides more robust results than convex-hull or similar estimates of geographical range that may be sensitive to varying environmental constraints, such as ice sheets and changing sea level. We collected these data for 18 time-slices representing subdivisions of North American land mammal ages (electronic supplementary material, table S2) and chose the time slice with the highest occupancy to represent maximum locality coverage for that species.

### 2.3. Statistics

To test for phylogenetic signal in the two intrinsic traits (body size, diet) and two emergent properties (duration, occupancy), we computed Pagel's *λ* [56] and Blomberg's *K* [57] using the R package phytools [58]. To account for phylogenetic signal if present, we conducted phylogenetic generalized least-squares (PGLS) analyses using the gls() function in the R package nlme [59], computing λ using maximum likelihood, and the best tree from the set of 500 fossil canid phylogenies generated by Slater [43] (electronic supplementary material, figure S4). We compared results with Brownian motion (*λ* = 1) and null (*λ* = 0) models using AICc and small-sample Akaike weights.

Preliminary observations showed a triangular relationship between our carnivory index and species longevity, with peak longevities occurring at mesocarnivorous values. To quantify the relationship on either side of the peak—for hypocarnivorous and hypercarnivorous taxa—we divided the data along the median carnivory value and tested for a linear correlation between longevity and carnivory for less and more carnivorous taxa separately.

A linear relationship between two variables may be obscured if variance in the sample varies with the independent variable (heteroscedasticity), possibly because a third unaccounted-for variable confounds the signal. Heteroscedasticity violates the constant-variance assumption of linear regression; therefore, linear regression is inappropriate to use in this case. Despite having refined our estimates of duration by factoring in sampling, the variance in duration values differs along the carnivory axis, with mesocarnivorous values having not only the highest longevities but also the greatest variation in longevity. The confounding variable is probably fossil preservation, which tends to vary with body size (larger species are more likely to be preserved than smaller species) and geographical range size (species dispersed more widely, in a variety of environments of differing likelihoods of preservation, tend to be more widely preserved than more localized species). To quantify the relationship between longevity and specialization for the best-preserved species—i.e. the relationship between maximum potential longevity and carnivory—we used quantile regression (R package quantreg [60]), which identifies the relationship between the response and predictor variables at certain quantiles (*τ*) [61–65]. In our case, linear regression (regression through the mean; *τ* = 0.5) provides a good estimate of duration when carnivory is close to the extremes (hypo- and hypercarnivory) and duration is restricted to low values; but, as diet approaches mesocarnivory, variance in duration increases, and carnivory loses power to predict duration in a linear regression model. However, even though regression at *τ* = 0.5 in this case does not provide useful information, regression at other quantiles may be meaningful. For this study, we modelled the slope of the relationship at *τ* = 0.6, 0.7, 0.8 and 0.9; *τ* = 0.9 represents the upper bound of the distribution, putatively when preservation is best. We bootstrapped the analysis for 10 000 replications to generate standard errors and *p*-values.

We explored the individual and combined effects of body mass, carnivory and maximum locality coverage on duration by running nested linear regressions on a subset of 77 fossil canid species for which we could record all four variables. We compared the nested models using AICc, Akaike weights and *R*^2^.

### 2.4. Models of trait evolution

To test if the evolution of body mass and carnivory in canids gravitated towards certain values (‘optima’) or progressed by other modes, we used maximum likelihood to fit six models of trait evolution to observed body-size and carnivory values, with best fit determined using small-sample Akaike weights. The six models include Brownian motion (BM), accelerating decelerating (ACDC), Trend, Drift, diversity dependence (Div) and Ornstein–Uhlenbeck (OU). BM is the null phylogenetic model, a random walk with no consistent trends; change is independent of past character states. ACDC refers to a rapid trait divergence at the beginning of a clade followed by slowed evolution towards the tips of the clade. Trend is BM incorporating a linear shift in evolutionary rate, while Drift is a trend towards larger or smaller trait values rather than rates. Div has rates varying as a function of past diversity [43], possibly resulting from competition increasing with species richness, which then propels trait convergence or divergence. Lastly, OU may be conceptualized as a ‘rubber band’ model: species may evolve away from ‘optimal’ values but would be constrained close to the optimum by the rubber band.

Slater [43] used a Bayesian fossil tip-dating approach on morphological data to generate a distribution of time-calibrated phylogenies for 121 canids, including extant and non-North American species. We pruned the phylogenies to the species also present in our fossil trait dataset (electronic supplementary material, figure S4). Slater's time calibration uses species stratigraphic ranges recorded from Wang *et al.* [35–37], closely reflecting our species temporal ranges inferred directly from occurrence data using PyRate and checked also using the monographs by Wang *et al*. Using the 500 canid phylogenies randomly sampled by Slater from the posterior distribution, we fit constant-rate BM, ACDC, Trend, Drift and OU models with the fitContinuous() function in the R package geiger [66], and the Div model with the fitDiversityModel() function in phytools [58]. Given previous work showing iterative occurrences of large body size and hypercarnivory within the three subfamilies [67], analysing traits on the family level may conflate iterative occurrences and obscure relationships between ecological traits and success; therefore, we also analysed trait evolution on subfamily trees extracted from the 500 randomly sampled phylogenies. We conducted statistical and phylogenetic analysis in R v. 3.4.1 [68].

## 3. Results

**Figure 1.**
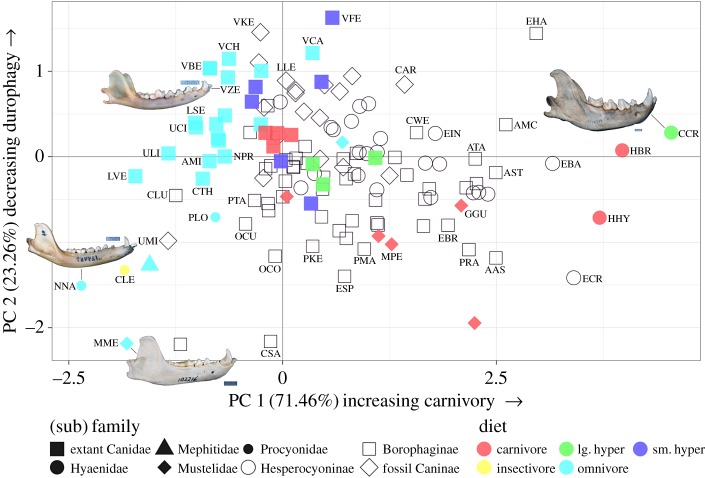
First two axes of principal component analysis run on three dietary indices of 45 extant caniform carnivorans and hyaenids, with extinct canids superimposed. ‘lg. hyper’ denotes large hypercarnivores; ‘sm. hyper’ denotes small hypercarnivores. Species are labelled where space permits. Representative images of lower jaws are included to illustrate extreme ecomorphologies (scaled to the same length; scale bars = 10 mm). Species abbreviations: AAS, *Aelurodon asthenostylus*; AMC, *Aelurodon mcgrewi*; AMI, *Atelocynus microtis*; AST, *Aelurodon stirtoni*; ATA, *Aelurodon taxoides*; CAR, *Canis armbrusteri*; CCR, *Crocuta crocuta*; CLU, *Cynarctoides luskensis*; CLE, *Conepatus leuconotus*; CSA, *Cynarctus saxatilis*; CTH, *Cerdocyon thous*; CWE, *Carpocyon webbi*; EBA, *Enhydrocyon basilatus*; EBR, *Euoplocyon brachygnathus*; ECR, *Enhydrocyon crassidens*; EIN, *Ectopocynus intermedius*; EHA, *Epicyon haydeni*; ESP, *Euoplocyon spissidens*; GGU, *Gulo gulo*; HBR, *Hyaena brunnea*; HHY, *Hyaena hyaena*; LLE, *Leptocyon leidyi*; LSE, *Lycalopex sechurae*; LVE, *Lycalopex vetulus*; MME, *Meles meles*; MPE, *Martes pennanti*; NNA, *Nasua nasua*; NPR, *Nyctereutes procyonoides*; OCO, *Otarocyon cooki*; OCU, *Oxetocyon cuspidatus*; PKE, *Paracynarctus kelloggi*; PLO, *Procyon lotor*; PMA, *Psalidocyon marianae*; PRA, *Protepicyon raki*; PTA, *Phlaocyon taylori*; UCI, *Urocyon cinereoargenteus*; ULI, *Urocyon littoralis*; UMI, *Urocyon minicephalus*; VBE, *Vulpes bengalensis*; VCA, *Vulpes cana*; VCH, *Vulpes chama*; VFE, *Vulpes ferrilata*; VKE, *Vulpes kernensis*; VZE, *Vulpes zerda*. Specimen images from Animal Diversity Web (http://www.animaldiversity.org/).

Figure 1 shows the first two axes of the principal component analysis run on the three dietary indices (electronic supplementary material, table S1) of the extant comparative dataset. Species that score highly on the first axis, such as the three extant hyaenids (*Crocuta crocuta*, *Hyaena brunnea* and *Hyaena hyaena*), have high relative blade length, deep jaws relative to dentary length, and small upper molar grinding areas relative to upper fourth premolar length. PC 1, therefore, ranges from less carnivorous on the left to more carnivorous on the right. PC 2 approximates durophagy—consumption of tough food items, such as bone—with less durophagous above and more durophagous below. Because the loading of RUGA (grinding area on the upper teeth relative to upper carnassial length) runs nearly parallel to PC 1 (electronic supplementary material, figure S5), it contributes little to PC 2; instead, PC 2 is driven by long blades on the positive side and deep jaws on the negative side.

The three dietary indices were preserved in 93 fossil canids of unknown diet (hollow shapes), whose predicted principal component values are superimposed onto the extant plot (figure 1). While most extant caniform carnivorans lie on the left of the plot, most fossil canids lie on the right, suggesting that fossil canids tend to have been more carnivorous and more durophagous than extant caniforms. In addition, the dietary diversity in fossil canids tends to surpass that of extant canids (filled squares, figure 1) and is shifted towards the robust morphologies of the wolverine (*Gulo gulo*) and the hyaenids.

**Figure 2.**
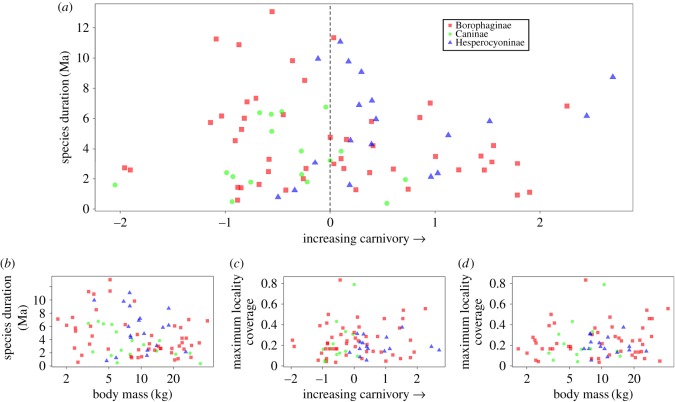
Bivariate plots of emergent properties against intrinsic traits of North American fossil canids. (*a*) Species duration (Ma) against carnivory increasing to the right. Carnivory is PC 1 re-centred around median = 0 (dashed line). Excepting a few outliers, the upper bounds of the data form a triangular shape. (*b*) Species duration against log_10_ body mass. (*c*) Maximum locality coverage against carnivory increasing to the right. (*d*) Maximum locality coverage against log_10_ body mass.

Rather than a linear correlation, a triangular pattern emerges when duration is plotted against carnivory index (figure 2*a*). Our dataset shows short durations occurring at values throughout the carnivory index; short durations are equally likely for less and more carnivorous canids. However, long durations occur only at mid-carnivory values. The upper left and upper right quadrants of figure 2*a*—the quadrants for long-lived hypo- and hypercarnivores, respectively—remain empty. There are three outliers on the hypercarnivorous side that are long-lived for their degree of specialization: the hesperocyonines *Enhydrocyon basilatus* (estimated duration = 6.16 Ma) and *Enhydrocyon crassidens* (8.73 Ma), and the borophagine *Epicyon haydeni* (6.83 Ma). These durations are still much shorter than the maximum duration, represented by the mesocarnivore *Cormocyon copei* at 13.07 Ma. A gap in carnivory values separates the rest of the canid distribution from the three most hypocarnivorous species, which are relatively short-lived: the borophagines *Cynarctus crucidens* (2.58 Ma) and *Cynarctoides luskensis* (2.72 Ma), and the canine *Urocyon minicephalus* (1.59 Ma).

Without correcting for phylogeny, there is a weak but significant negative relationship between body mass and species duration (figure 2*b*: *R*^2^ = 0.060, *p* = 0.013). However, this significance disappears after phylogenetic corrections (PGLS under BM with *λ* estimated by ML; *λ* = 0.853, *p* = 0.107). No linear relationship is apparent between carnivory and species duration, whether using raw trait values (*R*^2^ = 0.012, *p* = 0.942) or correcting for phylogeny (PGLS under BM with *λ* estimated by ML; *λ* = 0.879, *p* = 0.297). Maximum locality coverage also has no relationship with either body mass or carnivory (figure 2*c*: *R*^2^ = 0.009; *p* = 0.584; figure 2*d*: *R*^2^ = 0.002; *p* = 0.37).

**Figure 3.**
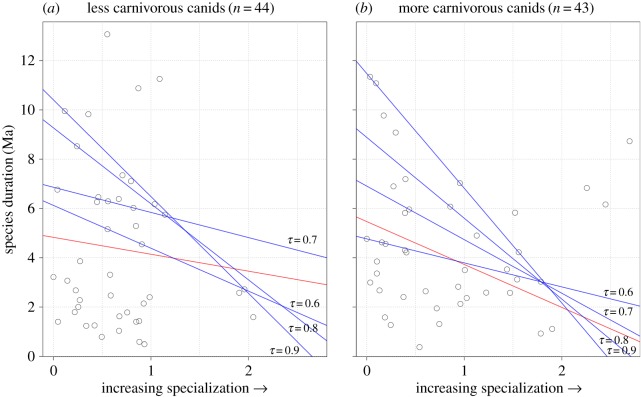
Relationship between species duration and specialization for less carnivorous **(***a***)** and more carnivorous **(***b***)** canids. The red line signifies an ordinary least-squares regression for less carnivorous canids and a phylogenetic generalized least-squares regression with *λ* = 0.538 for more carnivorous canids. Quantile regression lines in blue are shown for the 0.6, 0.7, 0.8 and 0.9 quantiles of duration versus specialization.

We analysed how species duration may vary with specialization on both dietary extremes (figure 3). The relationship between duration and specialization in less carnivorous taxa, having no phylogenetic signal, was analysed by an ordinary least-squares (OLS) regression; more carnivorous taxa, having moderate phylogenetic signal (*λ *= 0.538), was analysed by PGLS. On the more-carnivorous side, we excluded the three outlying hypercarnivorous species (*Enhydrocyon basilatus*, *Enhydrocyon crassidens* and *Epicyon haydeni*) from the regression analyses (resulting *n* = 40). The OLS result for lesser carnivory showed a negative association but no significant relationship between duration and hypocarnivorous specialization (*p* > 0.05); the PGLS result for greater carnivory showed a significant negative relationship between duration and hypercarnivorous specialization (*p* = 0.044).

We reinforced the standard regressions and accounted for heteroscedasticity by using quantile regressions. The relationship between duration and specialization for lesser carnivory is not significant at any quantile (*τ*), although duration tends to decrease as specialization approaches less carnivory; this tendency is strongest although still not significant at *τ* = 0.8 (table 2*a*; figure 3*a*). The tendency of duration to decrease with specialization for greater carnivory is more definitive: at *τ* ≥ 0.8, like the phylogenetic regression, there is a significant negative relationship between duration and greater carnivory (table 2*b*; figure 3*b*).

**Table 2.** Quantile regression results at four levels comparing species duration as a function of degree of specialization. All canids below the median carnivory value are less carnivorous (*a*); above the median, more carnivorous (*b*).

	intercept				slope			
quantile (*τ*)	value	s.e.	*t*	*p*	value	s.e.	*t*	*p*
(*a*) less carnivorous taxa								
0.6	6.127	1.854	3.304	0.002	−1.740	1.841	−0.945	0.350
0.7	6.863	1.700	4.036	0.000	−1.022	1.975	−0.517	0.608
0.8	9.274	1.869	4.963	0.000	−3.085	2.535	−1.217	0.230
0.9	10.409	1.983	5.248	0.000	−3.926	3.259	−1.205	0.235
(*b*) more carnivorous taxa								
0.6	4.767	1.297	3.677	0.001	−0.975	0.963	−1.012	0.318
0.7	6.908	1.749	3.949	0.000	−2.174	1.318	−1.649	0.107
0.8	8.884	1.792	4.960	0.000	−3.280	1.453	−2.257	0.030
0.9	11.474	1.336	8.591	0.000	−4.656	1.184	−3.933	0.000

**Figure 4.**
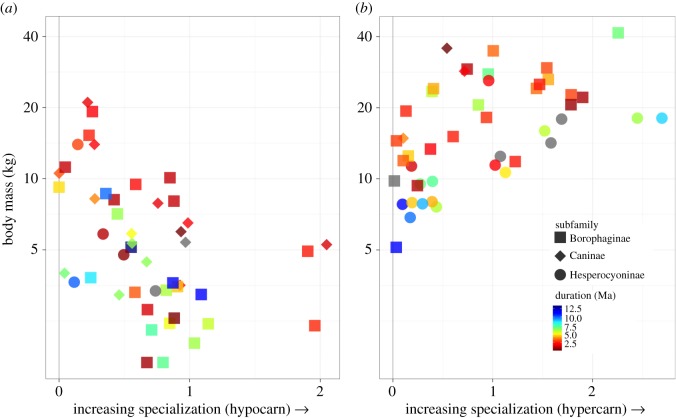
Bivariate plots visualizing species duration as a function of the combined effects of body size and specialization. Duration is mapped as colour. (*a*) For hypocarnivorous species; (*b*) for hypercarnivorous species.

Size and diet together—not as isolated traits—influence an animal's ecology, so it is not surprising that a model combining these two traits (logmass, carnivory) better explains species duration than does each of them separately (table 3; figure 4; hypercarnivorous outliers included). Visualizing duration colour-mapped onto bivariate plots of carnivory and body mass highlights key differences between hypocarnivory and hypercarnivory, the two extremes of dietary specialization examined here. The distribution defined by body mass, lesser degrees of carnivory and duration is straightforward: duration tends to decline with increasing mass and with increasing hypocarnivory (figure 4*a*). The distribution defined by body mass, greater degrees of carnivory and duration is more nuanced, showing longer durations at the highest carnivory values even as the prevailing trend is for decreased duration with higher carnivory (figure 4*b*) and suggesting that large body size combined with hypercarnivory biases species to shorter durations, to a point.

**Table 3.** Results from the nested linear regressions concerning effects of body mass, carnivory, maximum locality coverage and their combinations on the durations of 77 fossil canid species for which all variables have been recorded.

variable	loglk	AICc	AICw	Adj. *R*^2^	*p*
body mass only	−182.2848	368.7387	0.035979806	0.0511	0.02952
carnivory only	−184.7015	373.5720	0.003210084	−0.01295	0.7965
maxLocCover only	−182.8161	369.8013	0.021150124	0.03738	0.05409
body mass and carnivory	−178.0272	364.6341	0.280132853	0.1301	0.005135
body mass, carnivory and maxLocCover	−172.3531	362.9215	0.659527134	0.2085	0.00178

Could high occupancy allow a dietary specialist to last in the record longer, despite a heightened extinction risk that may be conferred by its specialization? Adding locality coverage to the interactive model as a proxy for dispersal ability increases the model's explanatory power (table 3). Despite high carnivory, species may endure when they have small to medium body size and wide geographical distribution: a combination of dietary specialization and geographical generalization.

**Figure 5.**
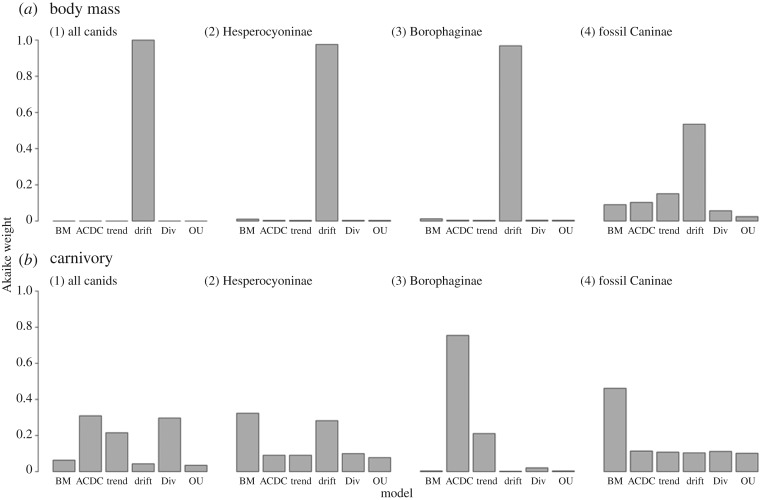
Median Akaike weights derived from model fits to 500 trees drawn randomly from the posterior distribution of trees. ‘Caninae’ in this sample includes only fossil North American Caninae. (*a*) Body mass; (*b*) carnivory.

The Ornstein–Uhlenbeck ‘rubber band’ model does not fit the evolution of mass or carnivory in the sampled canids. For the family Canidae sampled together, the best-supported model of *body mass* evolution is Drift, a directional shift in trait values (figure 5*a*). This shift is positive (electronic supplementary material, table S2), corroborating previous work documenting size increase in Canidae over time in a classic example of Cope's Rule [39]. Restricting the analysis to the subfamily level, the best-supported model of body mass evolution in both Hesperocyoninae and Borophaginae is again Drift towards larger body sizes; Drift is also the best-supported model of body mass evolution in fossil Caninae, although more equivocally than in the two extinct subfamilies (figure 5*a*). Including extant species in Caninae, no single model emerged to support the evolution of body mass. For the family Canidae sampled together, all tested models are poorly supported for the evolution of *carnivory* (figure 5*b*). Within Hesperocyoninae, the two best-supported models are BM and Drift. Within Borophaginae, ACDC is the best-supported model for the evolution of carnivory; the positive rate change parameter indicates an accelerating rate through time, or a late burst of evolution (electronic supplementary material, table S3). Within fossil Caninae, BM is marginally favoured over the other models (figure 5*b*), a result that also emerges when extant Caninae are included.

## 4. Discussion

Specific to canids, Van Valkenburgh *et al.* [39] showed a qualitative association, although no statistical correlation, between the evolution of large body size, a dietary shift to hypercarnivory, and a decline in species durations in Hesperocyoninae and Borophaginae, the two extinct subfamilies of North American canids. This lack of correlation stemmed, in part, from asymmetrical bias in the fossil record between short durations and long durations. Long durations are more verifiable as being long; short durations may be truly short or merely a signal of poor preservation. This asymmetry is visible as heteroscedasticity in the ‘filled triangle’ pattern emergent in the relationship between carnivory and duration (figure 2*a*), where a triangular upper bound is clearly delineated, but a lower bound is not. Focusing on the upper bound of the data using quantile regression enabled us to bypass this asymmetry.

*Body mass* alone, when corrected for phylogeny, was not implicated as a correlate of *duration*. However, the dietary measures examined tend to correlate with body mass (electronic supplementary material, figure S6) because prey size correlates with predator size. Fox-sized canids, for example, may be hypercarnivorous, but generally do not hunt prey larger than themselves, a distinction stemming from energetic requirements differing between species below and above approximately 21 kg [16,44]. Therefore, the jaws of fox-sized canids are proportioned less robustly than wolf-sized canids. Fox-sized canids also do not have the same bone-cracking adaptations as hyenas and hyena-like borophagine dogs [31], and consequently their jaws are relatively long and shallow rather than short and deep. While most of the measures were standardized to account for body mass (e.g. RBL is a measure of the lower slicing blade divided by lower carnassial length, our proxy for mass), the morphological differences between hunters of small versus large prey remain in proportions of linear traits, such as relative jaw depth.

Our analysis makes visible two patterns in the relationship between *duration* and *diet*: one for more carnivorous species and another for less carnivorous species. Rather than a simple linear correlation between duration and carnivory, the relationship is between duration and *specialization*: the more diet-specialized a species, either for greater or lesser carnivory, the shorter its duration in the fossil record is likely to be. While only the negative relationship between duration and hypercarnivorous specialization is significant, duration and hypocarnivory are still negatively associated. The lack of statistical support for this association may stem from the sparse record of species between the mesocarnivorous and hypocarnivorous range. Few species have values between one and two on the hypocarnivory scale (figure 3). This is probably because many putatively less-carnivorous species—e.g. several members of the genera *Cynarctoides* (4 of 8 species), *Leptocyon* (3 of 9), *Phlaocyon* (5 of 10) and *Urocyon* (3 of 5)*—*were preserved as fossils too fragmentary for calculation of the carnivory index. This poor preservation may itself suggest reduced persistence conferred by hypocarnivorous specialization.

Body size and dietary specialization were not correlated with *locality coverage* as estimated here. This result runs contrary to expectations that large species would have larger geographical ranges than small taxa because of better dispersal ability afforded by large body size, or that hypercarnivores would have larger ranges than hypocarnivores because meat is a constant resource not as restricted by environment as plant matter. Accurately quantifying geographical range is a particular challenge in the fossil record; other geographical measures such as abundance, or other methods of quantifying range, may provide better estimates of geographical success [6,54,55] and are currently being evaluated in a follow-up study. The moderate positive correlation between locality coverage and duration might reflect taphonomy, in that species preserved over longer timespans also may be preserved more broadly. However, it probably also represents a signal of biological success that might be resolved with better quality data: the same generalist traits that lead to longer durations might also lead to greater geographical coverage.

In modern ecosystems, species success is often defined as large geographical range, high population density, large group size and high reproductive rate. The interaction of these traits with each other complicates predictions of extinction risk [69]. Risk does not scale simply with body size; rather, complex interactions among correlated traits produce multiple pathways to extinction or persistence. For example, smaller species tend to have lower extinction risk than do larger species, in part because small mammalian body size correlates with large litter size and population size [70]. In the current analysis, smaller canid species also tend to live longer than larger canids, although the correlation disappears when corrected for phylogeny. Litter size is a trait that we are unable to measure in the fossil record, and thus remains an unmeasured possible covariate in our study.

We identified no movement towards a single evolutionarily optimal value for body size or carnivory across all Canidae. Rather, Cope's Rule—a phenomenon of body size increase within a lineage over time, well-documented among North American canids [39,71]—is echoed in our selection of the Drift model with a positive parameter for the body mass evolution of all canids, hesperocyonines only and borophagines only (figure 5; electronic supplementary material, table S3). This model is selected more ambiguously in fossil Caninae probably because of biogeographic differences: Caninae began to migrate outside North America approximately 7 Ma, while the two subfamilies remained endemic to the continent for the entirety of their durations [35–37]. It is possible that, had we not restricted our analyses to North America and instead included all fossil canids globally, we might have recovered an unambiguous Drift model, as with the two extinct subfamilies. However, the expansion of habitats available to Caninae upon migration probably impacted trait evolution in this clade.

For all Canidae and Hesperocyoninae only, no single model was best-supported for the evolution of carnivory. The conflation of different subfamily-level patterns probably caused the lack of resolution at the family level. Within Borophaginae, the best-supported model is ACDC, with a positive parameter indicating accelerating rate of evolution over time (electronic supplementary material, table S3). The selection of ACDC may be a statistical artefact: if traits evolved under constant-rate multivariate Brownian motion, but these traits were then reduced by standard rather than phylogenetic PCA as we have done, the first few principal component axes will appear to have evolved by an early burst process, a specific case of ACDC where rates decelerate through time [46]. Future studies of a multivariate trait such as carnivory would benefit from truly multivariate models of trait evolution. However, this result probably signals the directionality of the evolution of carnivory in Borophaginae: despite the early dominance of hypocarnivores in this subfamily, preliminary work shows that after approximately 16 Ma the subfamily shows a concerted movement towards hypercarnivory. The selection of Trend with a positive parameter—a linear increase in evolutionary rate—as the second-best model supports the primary selection of the ACDC model in suggesting increase in evolutionary rate of borophagine carnivory over time. Determining rate-shift points would represent a subsequent step towards identifying potential ecological drivers of the extreme dietary adaptations in this clade.

While examining abiotic influences, such as topographic complexity [72–74], is outside the scope of this study, research into the diversity dynamics of specialization would benefit from teasing apart possible interactions between abiotic and biotic processes in determining species success [4]. The spread of grasslands and opening of habitats after the Middle Miocene Climatic Optimum [75,76] probably impacted trait evolutionary rate, potentially accelerating the rise of large-bodied, bone-cracking and cursorial morphologies. Future work aims to test these hypotheses. The impact of congener competition on the ranges of young radiating lineages of mammalian carnivores, which our current dataset and methods lack the resolution to address, also presents an exciting opportunity for future study.

## 5. Conclusion

A negative relationship exists between species duration and dietary specialization, although no phylogenetically informed relationship exists between duration and body mass, between occupancy and body mass, and between occupancy and diet. Bone-cracking hypercarnivores tend to have shorter durations than small mesocarnivores, although we also identify an inflection point in the hypercarnivore morphospace beyond which some species appear to have surpassed constraints conferred by hypercarnivory. The negative relationship between duration and specialization may be mediated by geographical range and dispersal ability in an interplay between geographical range and taxonomic duration [77]: if they could disperse, even species of narrow resource use can be successful in time.

*Jack of all trades or master of one?* Our study of macroecological patterns in North American fossil canids provides evidence that small- to medium-sized species with more generalized diets persisted longer on average than both small hypocarnivores and large bone-cracking hypercarnivores. The apparently greater success of dietary generalists relative to specialists may result from the generalist ability to take advantage of a wider range of resources and thereby better withstand environmental and biotic perturbations.

## Supplementary Material

Main supplementary information document

## Data accessibility

The datasets and R code supporting this article have been uploaded as part of the electronic supplementary material at the Dryad Digital Repository: http://dx.doi.org/10.5061/dryad.1pj2600 [78].
