# Supplementary material for: Dietary specialization is linked to reduced species durations in North American fossil canids
Source: R Soc Open Sci. 2018 Apr 25;5(4):171861. doi: 10.1098/rsos.171861 (PMC5936914; doi:10.1098/rsos.171861)
Supplement: Main supplementary information document [file rsos171861supp1.pdf]

Supplementary Information

Table S1. Comparative database of 45 extant carnivoran species used to define the dietary morphospace. "Sm.hyper" denotes small (< 20 kg) hypercarnivore; lg.hyper denotes large (≥ 20 kg) hypercarnivore.

Family	Genus	Species	Mass (kg)	RBL	RUGA	JD/DL	Diet	Diet references
Canidae	*Vulpes*	*lagopus*	4.9	0.622	0.851	0.145	sm.hyper	[1–4]
Canidae	*Atelocynus*	*microtis*	9.5	0.608	1.127	0.136	omnivore	[5,6]
Canidae	*Canis*	*adustus*	9.5	0.611	1.032	0.128	omnivore	[7–11]
Canidae	*Canis*	*aureus*	9	0.639	0.949	0.143	carnivore	[8,12,13]
Canidae	*Canis*	*latrans*	14	0.643	0.847	0.14	carnivore	[14–18]
Canidae	*Canis*	*lupus*	51.5	0.646	0.862	0.166	lg.hyper	[19,20]
Canidae	*Canis*	*mesomelas*	7.5	0.642	0.906	0.138	carnivore	[8,11,13,21]
Canidae	*Canis*	*simensis*	15.25	0.664	1.006	0.122	sm.hyper	[22–24]
Canidae	*Cerdocyon*	*thous*	6.5	0.59	1.104	0.138	omnivore	[25–29]
Canidae	*Chrysocyon*	*brachyurus*	21.5	0.624	1.112	0.131	omnivore	[25,30]
Canidae	*Cuon*	*alpinus*	19	0.681	0.731	0.168	lg.hyper	[31,32]
Canidae	*Lycalopex*	*culpaeus*	8.7	0.633	0.905	0.13	omnivore	[24,33–37]
Canidae	*Lycalopex*	*griseus*	3	0.617	1.03	0.122	omnivore	[28,34,35,38]
Canidae	*Lycalopex*	*gymnocercus*	5.35	0.628	1.028	0.122	omnivore	[28,29,33]
Canidae	*Lycalopex*	*sechurae*	3.6	0.617	1.133	0.12	omnivore	[39]
Canidae	*Lycalopex*	*vetulus*	4	0.566	1.288	0.125	omnivore	[40,41]
Canidae	*Lycaon*	*pictus*	27	0.659	0.932	0.16	lg.hyper	[42–45]
Canidae	*Nyctereutes*	*procyonoides*	7	0.612	1.059	0.136	omnivore	[21,46,47]
Canidae	*Speothos*	*venaticus*	6	0.636	0.922	0.171	sm.hyper	[25,26,48]
Canidae	*Urocyon*	*cinereoargenteus*	4.5	0.613	1.119	0.121	omnivore	[49]
Canidae	*Urocyon*	*littoralis*	4	0.577	1.116	0.12	omnivore	[50–52]
Canidae	*Vulpes*	*bengalensis*	2.5	0.666	1.192	0.111	omnivore	[53,54]
Canidae	*Vulpes*	*cana*	0.8	0.712	0.91	0.125	omnivore	[55–57]
Canidae	*Vulpes*	*chama*	3	0.676	1.127	0.111	omnivore	[24,58]
Canidae	*Vulpes*	*corsac*	5	0.693	0.837	0.133	sm.hyper	[24,59]
Canidae	*Vulpes*	*ferrilata*	7	0.738	0.859	0.118	sm.hyper	[24,60]
Canidae	*Vulpes*	*macrotis*	2.1	0.643	0.94	0.122	sm.hyper	[24,61]
Canidae	*Vulpes*	*pallida*	2.8	0.656	1.129	0.108	omnivore	[24]

Canidae	*Vulpes*	*rueppellii*	3.3	0.677	1.007	0.119	omnivore	[62]
Canidae	*Vulpes*	*vulpes*	6	0.629	0.883	0.133	carnivore	[24,63]
Canidae	*Vulpes*	*zerda*	1.2	0.655	1.06	0.113	omnivore	[13,64]
Hyaenidae	*Crocuta*	*crocuta*	62.5	0.817	0	0.218	lg.hyper	[65–68]
Hyaenidae	*Hyaena*	*brunnea*	53.4	0.807	0.243	0.22	carnivore	[57,69–71]
Hyaenidae	*Hyaena*	*hyaena*	35	0.766	0.316	0.237	carnivore	[66]
Mephitidae	*Conepatus*	*leuconotus*	1.8	0.53	1.274	0.156	omnivore	[72]
Mustelidae	*Eira*	*barbara*	4.8	0.658	0.611	0.244	carnivore	[73,74]
Mustelidae	*Gulo*	*gulo*	21.5	0.685	0.472	0.197	carnivore	[75–77]
Mustelidae	*Martes*	*pennanti*	3.4	0.642	0.68	0.197	carnivore	[78]
Mustelidae	*Meles*	*meles*	13	0.514	1.541	0.186	omnivore	[79–81]
Mustelidae	*Mellivora*	*capensis*	10.3	0.603	0.475	0.18	carnivore	[7,82,83]
Mustelidae	*Melogale*	*moschata*	2	0.647	0.635	0.148	omnivore	[84,85]
Mustelidae	*Taxidea*	*taxus*	8.4	0.629	0.979	0.164	carnivore	[57,72]
Procyonidae	*Nasua*	*narica*	5	0.531	1.424	0.157	insectivore	[86,87]
Procyonidae	*Nasua*	*nasua*	4.6	0.469	1.304	0.142	omnivore	[57,88]
Procyonidae	*Procyon*	*lotor*	6.5	0.632	1.427	0.17	omnivore	[57,72]

Table S2. Time intervals used for analysis of maximum geographic range area. Intervals follow the age bounds defined on the MIOMAP / FAUNMAP database, except for the Early / Late Clarendonian pair of intervals, which were assigned to be of equal size after preliminary analysis showed that using MIOMAP's original Early / Middle / Late Clarendonian subdivisions produced extreme unevenness in number of localities across the Clarendonian.

Interval name	Abbreviation	Lower bound (million years ago)	Upper bound (million years ago)
Early Early Arikareean	EEAK	30	27.9
Late Early Arikareean	LEAK	27.9	23.8
Early Late Arikareean	ELAK	23.8	19.5
Late Late Arikareean	LLAK	19.5	18.8
Early Hemingfordian	EHMF	18.8	17.5
Late Hemingfordian	LHMF	17.5	15.9
Early Barstovian	EBAR	15.9	14.8
Late Barstovian	LBAR	14.8	12.5
Early Clarendonian	ECLA	12.5	10.75
Late Clarendonian	LCLA	10.75	9
Early Early Hemphillian	EEHP	9	7.5
Late Early Hemphillian	LEHP	7.5	6.7
Early Late Hemphillian	ELHP	6.7	5.9
Late Late Hemphillian	LLHP	5.9	4.7
Blancan	BLAN	4.7	1.7
Irvingtonian	IRVI	1.7	0.45
Rancholabrean	RANC	0.45	0.01
Holocene	HOLO	0.01	0

Table S3. Median results from models of trait evolution fitted to 500 trees sampled at random from the Bayesian posterior distribution by Slater [89].

Model	Log likelihood	AICc	AICw	σ^2	parameter
**A. All canids**					
**1. Body mass**					
BM	44.66475	-85.21069	9.157756e-05	0.003137757	NA
ACDC	44.82939	-83.41878	3.961611e-05	0.002973134	2.162607e-03
Trend	44.19477	-82.14953	3.065730e-05	0.002961913	2.894189e-03
Drift	54.88301	-103.52601	9.997347e-01	0.002574601	1.827889e-02
Diversity	44.91393	-83.58786	4.295418e-05	0.002830693	6.421031e-06
OU	44.66091	-83.08183	3.446327e-05	0.003138818	5.638945e-11
**2. Carnivory**					
BM	-85.58034	175.3145	0.06286337	0.05542281	NA
ACDC	-82.93337	172.1784	0.30904457	0.02338048	3.757759e-02
Trend	-83.59806	173.5078	0.21499314	0.01879732	2.894189e-03
Drift	-84.89842	176.1085	0.04285114	0.05429989	2.199654e-02
Diversity	-82.93179	172.1753	0.29672574	0.03478219	4.402561e-04
OU	-85.15941	176.6305	0.03487011	0.06291428	5.638945e-11
**B. Hesperocyoninae**					
**1. Body mass**					
BM	10.72104	-16.89664	0.010247768	0.002784072	NA
ACDC	10.90481	-14.66677	0.003364927	0.003324918	-1.554880e-02
Trend	10.69501	-14.24717	0.002814115	0.003080303	-8.770118e-03
Drift	16.56953	-25.99621	0.975773477	0.001726929	2.229475e-02
Diversity	10.92228	-14.70171	0.003365869	0.003019731	-1.813923e-05
OU	10.73479	-14.32673	0.002868430	0.003019731	4.874302e-11
**2. Carnivory**					
BM	-16.80593	38.41185	0.32331540	0.03979974	NA
ACDC	-16.57325	40.86079	0.09059419	0.04690875	-1.791413e-02
Trend	-16.74146	41.19720	0.09059419	0.04389444	-8.770118e-03
Drift	-15.43745	38.58919	0.28213640	0.03413788	4.820888e-02
Diversity	-16.50592	40.72612	0.09957292	0.03664228	4.999463e-04
OU	-16.81839	41.35107	0.07754502	0.03981831	4.874302e-11
**C. Borophaginae**					
**1. Body mass**					
BM	24.40350	-44.58059	0.012110790	0.003053074	NA
ACDC	24.49023	-42.51892	0.004613334	0.002941919	1.023808e-03
Trend	24.06219	-41.66283	0.003805079	0.002739413	4.655062e-03
Drift	29.88019	-53.29883	0.968437135	0.002526532	2.142011e-02
Diversity	24.55110	-42.64065	0.004800221	0.002730486	1.277821e-05
OU	24.40370	-42.34586	0.004210236	0.003049184	3.025531e-11
**2. Carnivory**					
BM	-53.39299	111.05871	0.003421490	6.526654e-02	NA

ACDC	-46.60766	99.77347	0.754624015	4.906135e-03	1.062904e-01
Trend	-48.03736	102.63287	0.210585899	2.675498e-05	3.488621e-03
Drift	-52.81768	112.19350	0.001821449	6.379503e-02	3.459980e-02
Diversity	-50.36962	107.29738	0.020662595	3.881006e-02	8.624105e-04
OU	-52.30791	111.17395	0.003301093	8.701984e-02	4.405020e-11
**D. Fossil Caninae**					
**1. Body mass**					
BM	13.76979	-22.93958	0.09041973	2.941388e-03	NA
ACDC	14.99181	-22.72046	0.10259634	7.382779e-04	6.495250e-02
Trend	15.35436	-23.44556	0.15091620	4.799969e-06	5.096073e+01
Drift	16.86994	-26.47673	0.53512705	2.244663e-03	1.748150e-02
Diversity	14.52832	-21.79348	0.05662464	1.877707e-03	8.941093e-05
OU	13.76245	-20.26174	0.02399209	2.938599e-03	4.348186e-11
**2. Carnivory**					
BM	-11.22622	27.37552	0.4616176	0.03449126	NA
ACDC	-11.05996	30.11993	0.1138682	0.02490613	1.662639e-02
Trend	-11.19122	30.38243	0.1077072	0.02484354	5.096073e+01
Drift	-11.17205	30.34409	0.1037565	0.03419290	-2.057593e-03
Diversity	-11.08674	30.17349	0.1116702	0.02626673	1.116238e-03
OU	-11.21163	30.42325	0.1013317	0.03648666	4.348186e-11

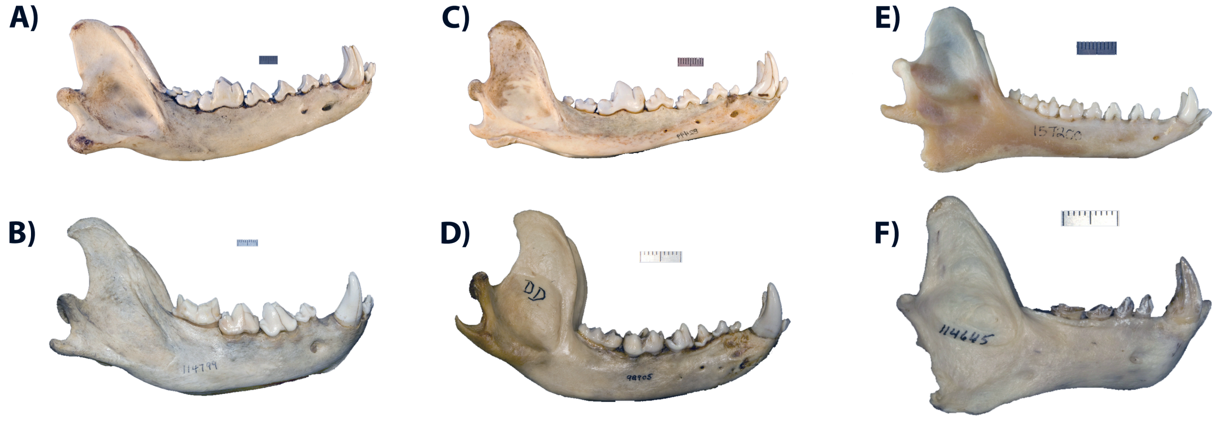

Figure S1. Lower jaws of representative extant carnivoran species across the hypercarnivory-mesocarnivory-hypocarnivory spectrum. **A, B,** hypercarnivores; **C, D,** mesocarnivores; **E, F,** hypocarnivores. **A,** grey wolf, *Canis lupus*; **B,** spotted hyena, *Crocuta crocuta*; **C,** coyote, *Canis latrans*; **D,** raccoon, *Procyon lotor*; **E,** bat-eared fox, *Otocyon megalotis*; **F,** kinkajou, *Potos flavus*. To highlight the differences in skeletal proportions corresponding to functional and dietary divergence among these animals, the lower jaws have been scaled to the same length from mandibular condyle to anterior end. Scale bars = 10 mm. Original specimen photos from Animal Diversity Web: https://animaldiversity.org.

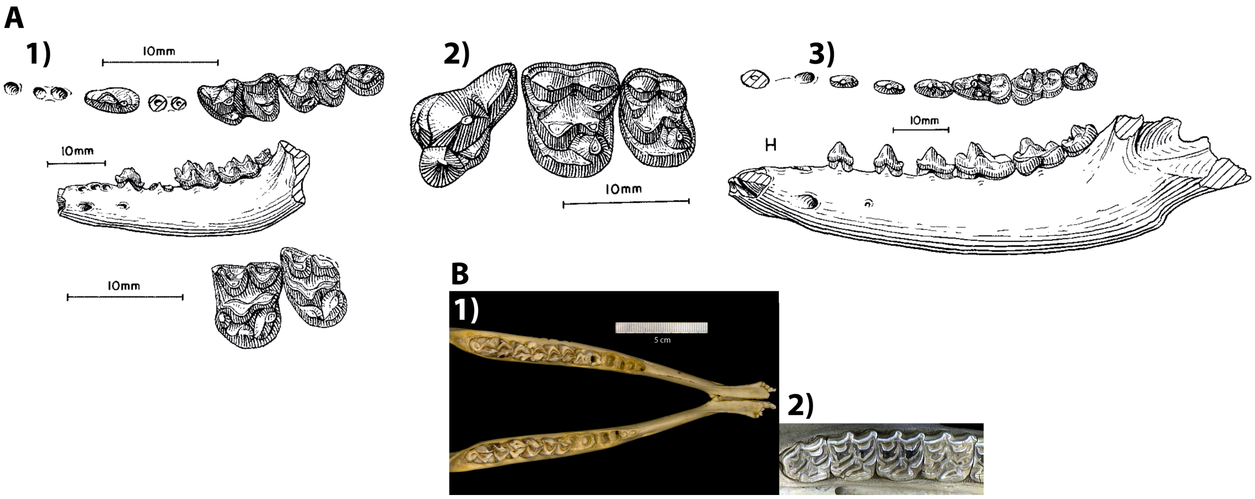

Figure S2. Comparison of the dentition of putatively hypocarnivorous fossil canids **(A)** with the dentition of known extant herbivorous ungulates **(B)**. **A, 1)** *Cynarctoides emryi*: occlusal view of lower dentition (top), labial view of lower jaw (middle), and occlusal view of upper molars (bottom); **2)** *Phlaocyon achoros*: occlusal view of upper carnassial and molars; **3)** *Cynarctus crucidens*, occlusal view of lower dentition (top) and labial view of lower jaw (bottom). **B, 1)** occlusal view of lower jaw of impala, *Aepyceros melampus*, and 2) occlusal view of dentition of horse, *Equus caballus*. Putatively hypocarnivorous fossil canids exhibit ecomorphological specialization in their teeth, with dental wear creating ridges converging on ungulate selenodont or bunodont dental morphology. Fossil-canid illustrations are from Wang *et al.* [90] and included with permission; extant-ungulate specimen photographs are from Animal Diversity Web (https://animaldiversity.org).

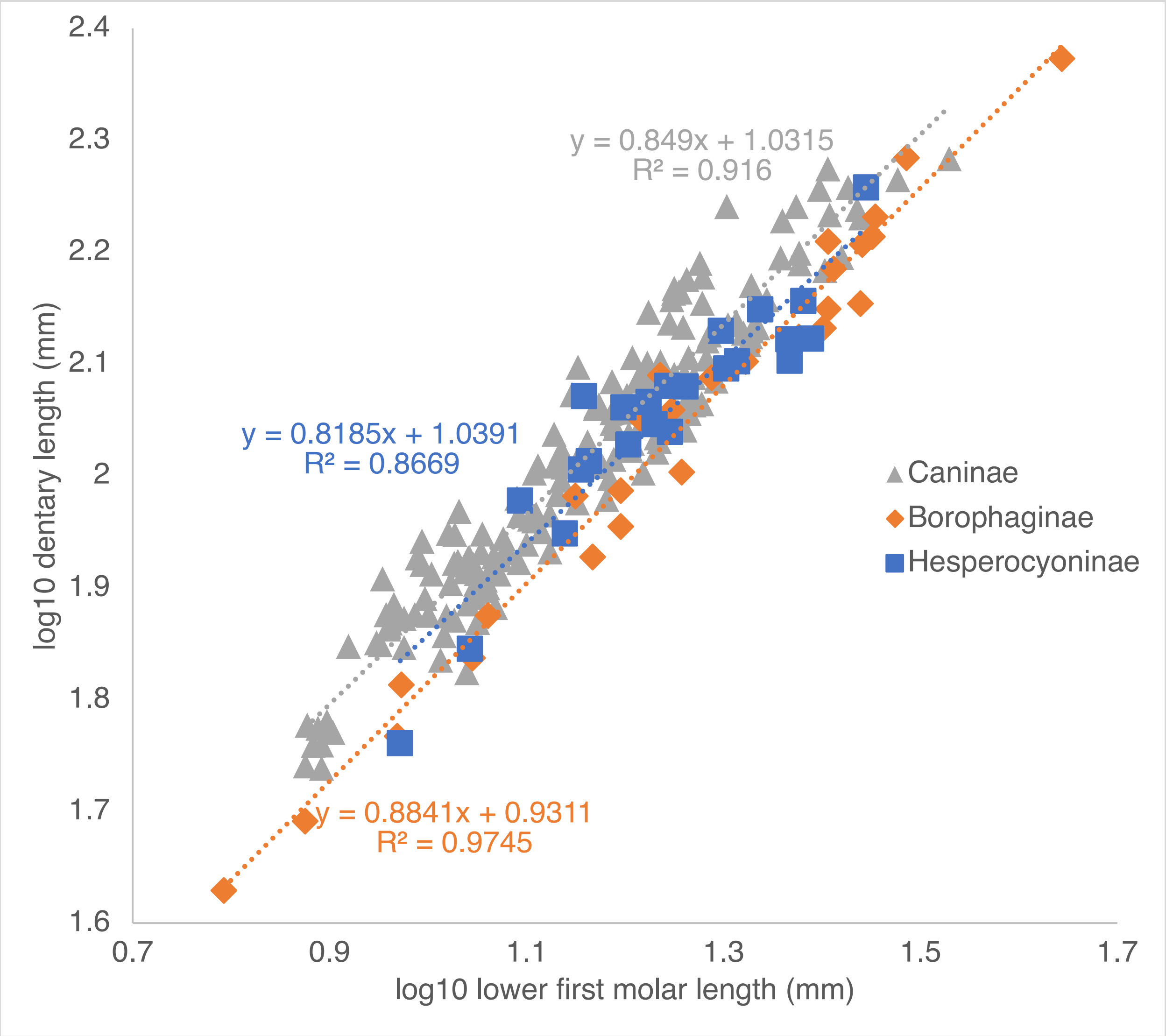

Figure S3. Per-subfamily regressions of $\log_{10}$ dentary length on $\log_{10}$ lower first molar length used to estimate dentary length of fragmentary fossil species, based on intact dentaries of 24 hesperocyonine individuals, 27 borophagine individuals, and 175 canine (extinct and extant) individuals. Borophagine dentaries tend to be shorter for a given molar length than dentaries of other subfamilies, even for borophagines of smaller body size that would not be expected to use a robust dentary to process bone.

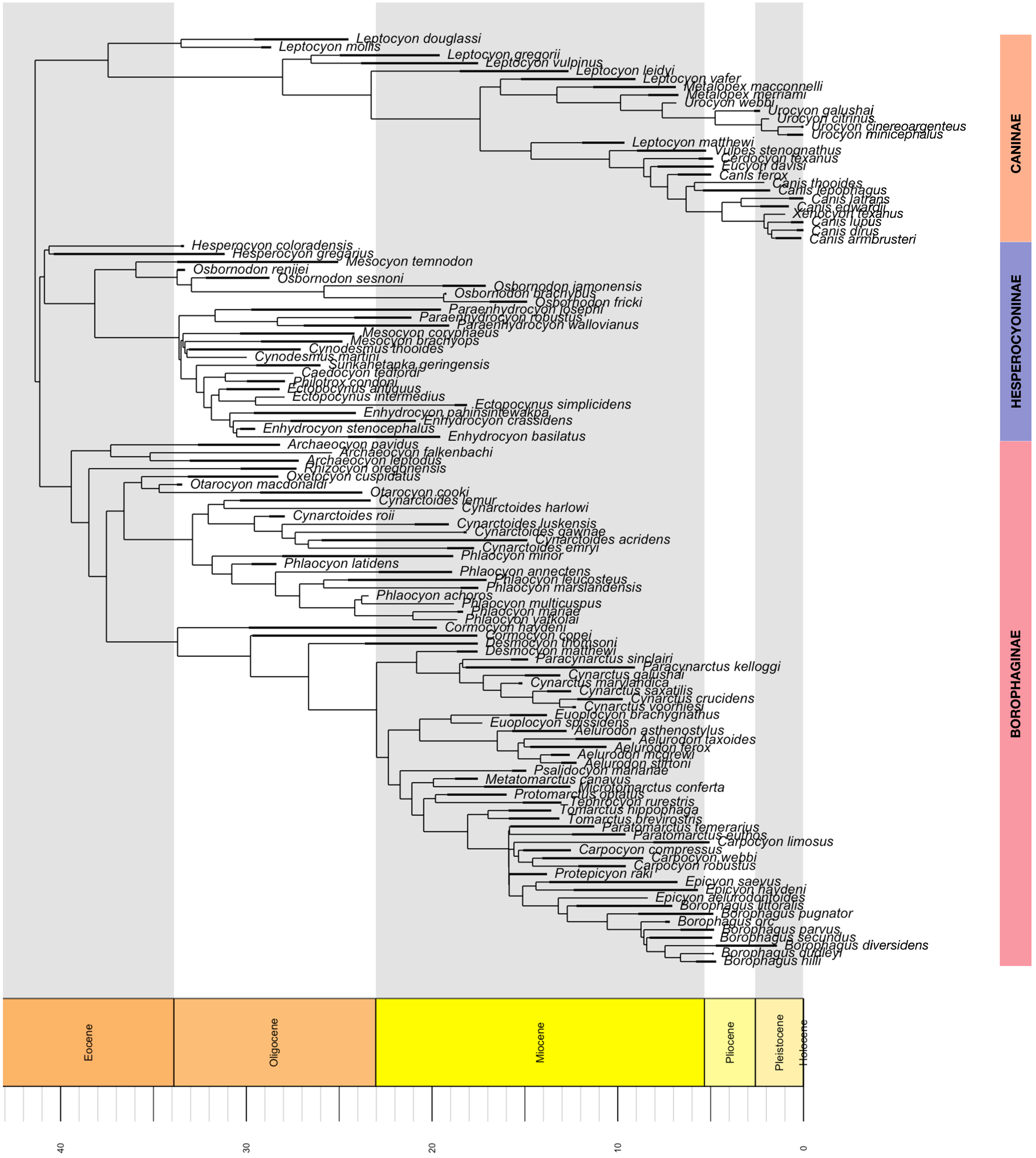

Figure S4. Time-scaled canid tree; topology from tree with the highest log-likelihood from the Bayesian posterior probability distribution of Slater [89]. Black bars on branches indicate stratigraphic range recorded by occurrences. Branches lacking black bars indicate singletons, or species with only one occurrence. Time units are millions of years ago.

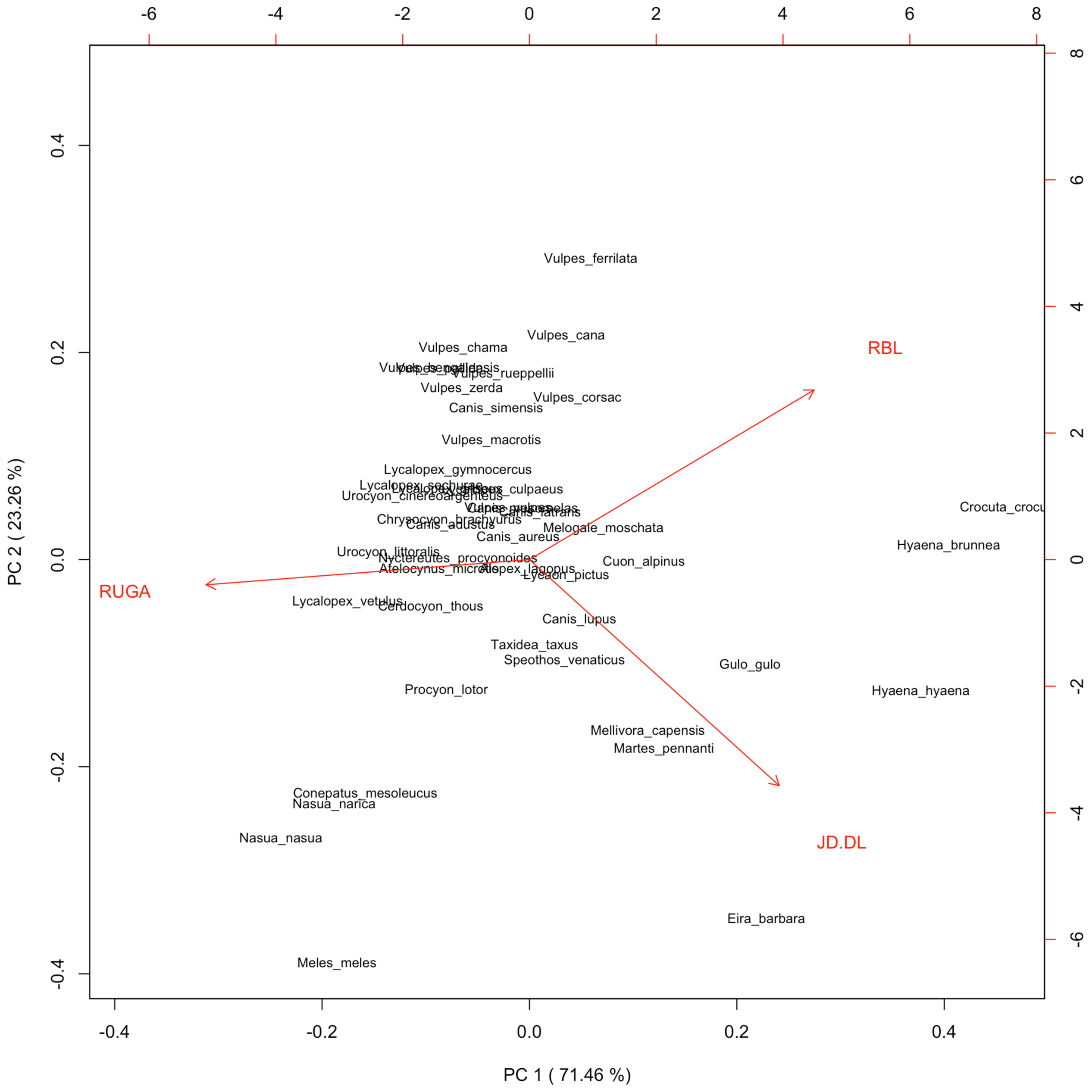

Figure S5. Principal component analysis of 45 extant caniform and hyaenid species only, with direction and magnitude of loadings superimposed.

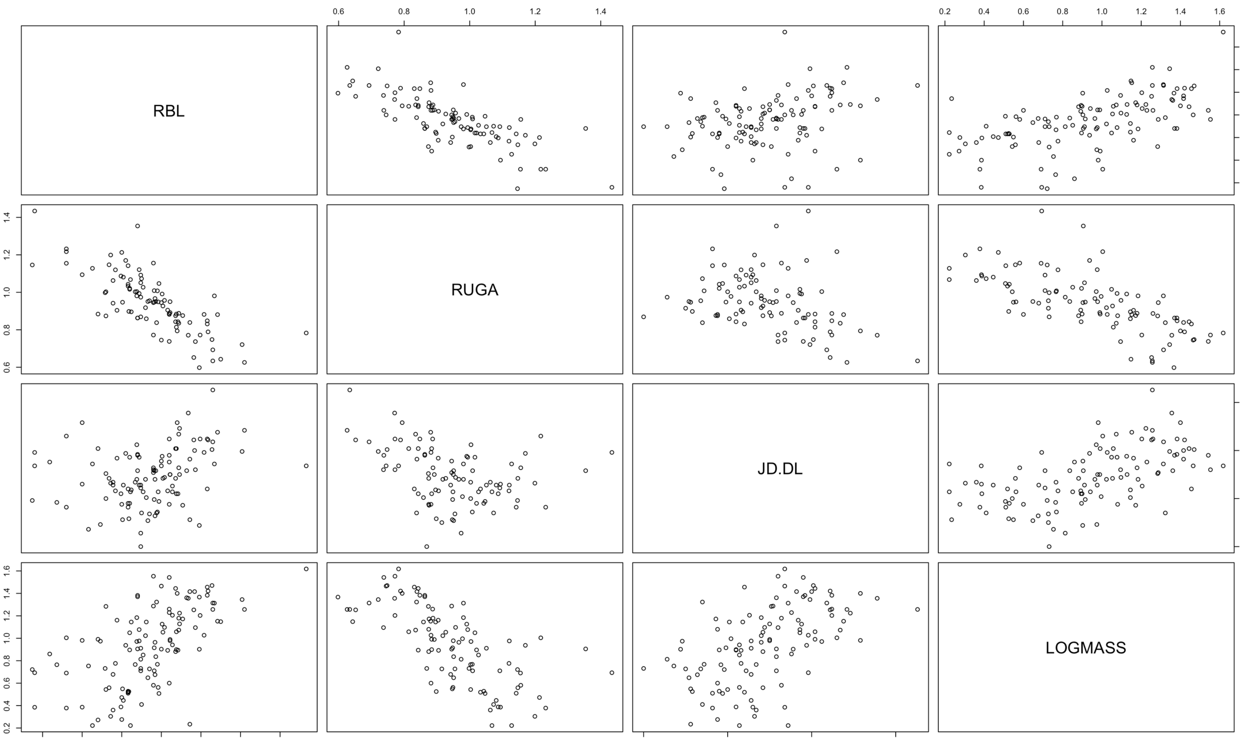

Figure S6. Bivariate plots showing correlated patterns among dietary measures and body mass.

Supplementary References

1. Garrott, R. A. & Eberhardt, L. E. 1987 Arctic fox. In *Wild furbearer management and conservation in North America* (eds M. Novak J. A. Baker M. E. Obbard & B. Malloch), pp. 394–406. Ontario: Ministry of Natural Resources.
2. Angerbjörn, A., Tannerfeldt, M. & Erlinge, S. 1999 Predator-prey relationships: Arctic foxes and lemmings. *J. Anim. Ecol.* **68**, 34–49. (doi:10.1046/j.1365-2656.1999.00258.x)
3. Elmhagen, B., Tannerfeldt, M., Verucci, P. & Angerbjörn, A. 2000 The arctic fox (Alopex lagopus): an opportunistic specialist. *J. Zool.* **251**, 139–149. (doi:10.1111/j.1469-7998.2000.tb00599.x)
4. Kaikusalo, A. & Angerbjörn, A. 1995 The arctic fox population in Finnish Lapland during 30 years, 1964-93. *Ann. Zool. Fennici* **32**, 69–77.
5. Berta, A. 1986 Atelocynus microtis. *Mamm. Species* , 1–3. (doi:10.1126/science.95.2469.427-b)
6. Leite Pitman, M. R. P. & Williams, R. S. R. 2004 The short eared dog (Atelocynus microtis). *Canids Species status Conserv. Action Plan*.
7. Rosevear, D. R. 1974 *The carnivores of west Africa*. London: Trustees of the British Museum (Natural History).
8. Lamprecht, J. 1978 On diet, foraging behavior and interspecific food competition of jackals in the Serengeti National Park, east Africa. *Zeitschrift für Säugetierkd.* **43**, 210–223.
9. Atkinson, R. P. D., Macdonald, D. W. & Kamizola, R. 2002 Dietary opportunism in side-striped jackals Canis adustus Sundevall. *J. Zool.* **257**, 129–139. (doi:Doi 10.1017/S0952836902000729)
10. Loveridge, A. J. & Macdonald, D. W. 2003 Niche separation in sympatric jackals (Canis mesomelas and Canis adustus). *J. Zool.* **259**, S0952836902003114. (doi:10.1017/S0952836902003114)
11. Skinner, J. D. & Chimimba, C. T. 2005 *The mammals of the southern African sub-region*. Cambridge University Press.
12. Hufnagl, E., Craig-Bennett, A., Brogan, O., Savage, R. J. G. & van Weerd, E. 1972 *Libyan mammals*. Oleander Press.
13. Kingdon, J. 1977 IIIA. Carnivores. In *East African mammals: an atlas of evolution in Africa*, pp. 491. University of Chicago Press.
14. Murie, A. 1940 Ecology of the coyote in the Yellowstone. *Fauna Natl. Park. United States*. **4**.
15. Ferrel, C. M., Leach, H. R. & Tillotson, D. F. 1953 Food habits of the coyote in California. *Calif. Fish Game*. **39**.
16. Berg, W. E. & Chesness, R. A. 1978 Ecology of coyotes in northern Minnesota. In *Coyotes: biology, behavior, and management* (ed M. Bekoff), pp. 229–247. New York: Academic Press.
17. Bowen, W. D. 1981 Variation in coyote social organization: the influence of prey size. *Can. J. Zool.* **59**, 639–652. (doi:10.1139/z81-094)
18. Wells, M. C. & Bekoff, M. 1982 Predation by wild coyotes: behavioral and ecological analyses. *J. Mammal.* **63**, 118–127.

19. Peterson, R. O. 1974 Wolf ecology and prey relationships on Isle Royale. *United States Natl. Park Serv. Sci. Monogr. Ser.* **11**, 1–210.
20. Mech, L. D. & Boitani, L. 2003 *Wolves: behavior, ecology, and conservation*. University of Chicago Press.
21. Colby, C. B. 1965 *Wild dogs*. New York, NY: Duell, Sloane and Pearce.
22. Sillero-Zubiri, C. & Gottelli, D. 1994 Canis simensis. *Mamm. Species* , 1–6. (doi:10.2307/3504136)
23. Malcolm, J. 1997 The diet of the Ethopian wolf (Canis simensis Ruppell) from a grassland area of the Bale Mountains, Ethiopia. *Afr. J. Ecol.* **35**, 162–164. (doi:10.1046/j.1365-2028.1999.00148.x)
24. Sillero-Zubiri, C., Hoffmann, M. & Macdonald, D. W. 2004 Canids: Foxes, Wolves, Jackals and Dogs.
25. Langguth, A. 1975 Ecology and evolution in the South American canids. In *The wild canids: their systematics, behavioral ecology, and evolution* (ed M. W. Fox), pp. 192–206. New York: Van Nostrand Reinhold Company.
26. Husson, A. M. 1978 *The mammals of Suriname*.
27. Berta, A. 1982 Cerdocyon thous. *Mamm. Species* , 1–4.
28. Medel, R. G. & Jaksić, F. M. 1988 Ecología de los cánidos sudamericanos: una revisión. *Rev. Chil. Hist. Nat.* **61**, 67–79.
29. Mares, M. A., Ojeda, R. A. & Barquez, R. M. 1989 *Guide to the mammals of Salta Province, Argentina*. Norman: University of Oklahoma Press.
30. Dietz, J. M. 1985 Chrysocyon brachyurus. *Mamm. Species* , 1–4.
31. Johnsingh, A. J. T. 1981 Ecology and behavior of the dhole, Cuon alpinus, in India.
32. Fox, M. W. 1985 *The whistling hunters: field studies of the Asiatic wild dog (Cuon alpinus)*. State University of New York Press.
33. Crespo, J. A. 1975 Ecology of the pampas gray fox and the large fox (culpeo). In *The wild canids: their systematics, behavioral ecology, and evolution* (ed M. W. Fox), pp. 179–191. New York, NY: Van Nostrand Reinhold Company.
34. Fuentes, E. R. & Jaksić, F. M. 1979 Latitudinal size variation of Chilean foxes: tests of alternative hypotheses. *Ecology* **60**, 43–47.
35. Jaksić, F. M., Schlatter, R. P. & Yáñez, J. L. 1980 Feeding ecology of central Chilean foxes, Dusicyon culpaeus and Dusicyon griseus. *J. Mammal.* **61**, 254–260.
36. Novaro, A. J. 1997 Pseudalopex culpaeus. *Mamm. species* , 1–8.
37. Walker, R. S., Novaro, A. J., Perovic, P., Palacios, R., Donadio, E., Lucherini, M., Pia, M. & López, M. S. 2007 Diets of three species of Andean carnivores in high-altitude deserts of Argentina. *J. Mammal.* **88**, 519–525. (doi:10.1644/06-MAMM-A-172R.1)
38. Jaksić, F. M. & Yáñez, J. L. 1983 Rabbit and fox introductions in Tierra del Fuego: history and assessment of the attempts at biological control of the rabbit infestation. *Biol. Conserv.* **26**, 367–374. (doi:10.1016/0006-3207(83)90097-6)
39. Huey, R. B. 1969 Winter diet of the Peruvian desert fox. *Ecology* **50**, 1089–1091.
40. Dalponte, J. C. 1997 Diet of the hoary fox, Lycalopex vetulus, in Mato Grosso, central Brazil. *Mammalia* **61**, 537–546.
41. Eisenberg, J. F. & Redford, K. H. 2000 *Mammals of the neotropics*. University of Chicago Press.
42. Estes, R. D. & Goddard, J. 1967 Prey selection and hunting behavior of the African wild

dog. *J. Wildl. Manage.* **31**, 52–70.
43. Kruuk, H. & Turner, M. 1967 Comparative notes on predation by lion, leopard, cheetah and wild dog in the Serengeti area, east Africa. *Mammalia* , 1–27.
44. Kruuk, H. 1972 Surplus killing by carnivores. *J. Zool.* **166**, 233–244.
45. Schaller, G. B. 1972 *The Serengeti lion: a study of predator-prey relations*. University of Chicago Press.
46. Ward, O. G. & Wurster-Hill, D. H. 1989 Ecological studies of Japanese raccoon dogs, Nyctereutes procyonoides viverrinus. *J. Mammal.* **70**, 330–334.
47. Sheldon, J. W. 2004 *Wild dogs: The natural history of the nondomestic Canidae*. Caldwell, N.J.: Blackburn Press.
48. Deutsch, L. A. 1983 An encounter between bush dog (Speothos venaticus) and paca (Agouti paca). *J. Mammal.* **64**, 532–533.
49. Fritzell, E. K. & Haroldson, K. J. 1982 Urocyon cinereoargenteus. *Mamm. Species* , 1–8.
50. Collins, P. W. 1991 Interaction between island foxes (Urocyon littoralis) and Indians on islands off the coast of Southern California: I. Morphologic and archaeological evidence of human assisted dispersal. *J. Ethnobiol.* **11**, 51–81.
51. Moore, C. M. & Collins, P. W. 1995 Urocyon littoralis. *Mamm. Species* , 1–7.
52. Garcelon, D. K., Roemer, G. W., Philips, R. B. & Coonan, T. J. 1999 Food provisioning by island foxes, Urocyon littoralis, to conspecifics caught in traps. *Southwest. Nat.* **44**, 83–86.
53. Johnsingh, A. J. T. 1978 Some aspects of the ecology and behaviour of the Indian fox Vulpes bengalensis (Shaw). *J. Bombay Nat. Hist. Soc.* **106**, 397–405.
54. Manakadan, R. & Rahmani, A. R. 2000 Population and ecology of the Indian fox Vulpes bengalensis at Rollapadu Wildlife Sanctuary, Andhra Pradesh, India. *J. Bombay Nat. Hist. Soc.* **97**, 3–14.
55. Geffen, E., Hefner, R., MacDonald, D. W. & Ucko, M. 1992 Diet and foraging behavior of Blanford's foxes, Vulpes cana, in Israel. *J. Mammal.* **73**, 395–402. (doi:10.2307/1382074)
56. Geffen, E. & Macdonald, D. W. 1992 Small size and monogamy: Spatial organization of Blanford's foxes, Vulpes cana. *Anim. Behav.* **44**, 1123–1130. (doi:10.1016/s0003-3472(05)80324-0)
57. Nowak, R. M. 1999 *Walker's Mammals of the World*. 6th edn. Johns Hopkins University Press.
58. Nel, J. A. J. 1984 Behavioural ecology of canids in the southwestern Kalahari. *Koedoe* **27**, 229–235.
59. Clark Jr, H. O., Murdoch, J. D., Newman, D. P. & Sillero-Zubiri, C. 2009 Vulpes corsac (Carnivora: Canidae). *Mamm. Species* , 1–8. (doi:10.1644/832.1.Key)
60. Schaller, G. B. 1998 *Wildlife of the Tibetan steppe*. University of Chicago Press.
61. Meaney, C. A., Reed-Eckert, M. & Beauvais, G. P. 2006 Kit fox (Vulpes macrotis): A technical conservation assessment.
62. Larivière, S. & Seddon, P. J. 2001 Vulpes rueppelli. *Mamm. Species* **678**, 1–5. (doi:10.1644/1545-1410(2001)678<0001:VR>2.0.CO;2)
63. Larivière, S. & Pasitschniak-Arts, M. 1996 Vulpes vulpes. *Mamm. Species* , 1–11. (doi:10.2307/3504236/2600732)
64. Osborn, D. & Osbornova, J. 1998 *The mammals of ancient Egypt*. Aris and Phillips.
65. Kruuk, H. 1972 *The spotted hyena: a study of predation and social behavior*. Chicago:

University of Chicago Press.
66. Mills, G. & Hofer, H. 1998 Hyaenas: Status survey and conservation action plan.
67. Honer, O. P., Wachter, B., East, M. L. & Hofer, H. 2002 The response of spotted hyaenas to long-term changes in prey populations: Functional response and interspecific kleptoparasitism. *J. Anim. Ecol.* **71**, 236–246.
68. Hofer, H. & East, M. L. 2003 Behavioral processes and cost of co-existence in female spotted hyenas: a life history perspective. *Evol. Ecol.* **17**, 315–331. (doi:10.1023/A:1027352517231)
69. Owens, D. D. & Owens, M. J. 1979 Communal denning and clan associations in brown hyenas (Hyaena brunnea, Thunberg) of the central Kalahari Desert. *Afr. J. Ecol.* **17**, 35–44. (doi:10.1111/j.1365-2028.1979.tb00454.x)
70. Skinner, J. D. & van Aarde, R. J. 1991 Bone collecting by brown hyaenas Hyaena brunnea in the central Namib Desert, Namibia. *J. Archaeol. Sci.* **18**, 513–523. (doi:10.1016/0305-4403(91)90051-P)
71. Maude, G. 2005 The comparative ecology of the Brown Hyaena (Hyaena brunnea) in Makgadikgadi National Park and a Neighbouring Community Cattle Area in Botswana.
72. Wilson, D. E. & Ruff, S., editors 1999 *The Smithsonian Book of North American Mammals*. Washington, DC: Smithsonian Institution Press.
73. Bisbal E, F. J. 1986 Food habits of some neotropical carnivores in Venezuela (Mammalia, Carnivora). *Mammalia* **50**, 329–340.
74. McNab, B. K. 1995 Energy expenditure and conservation in frugivorous and mixed-diet carnivorans. *J. Mammal.* **76**, 206–222.
75. Burt, W. H. 1948 *The mammals of Michigan*. Ann Arbor, MI: University of Michigan Press.
76. Pasitschniak-Arts, M. & Larivière, S. 1995 Gulo gulo. *Mamm. Species* , 1–10. (doi:10.1644/0.499.1/2600717)
77. Lofroth, E. C., Krebs, J. A., Harrower, W. L. & Lewis, D. 2007 Food habits of wolverine Gulo gulo in montane ecosystems of British Columbia, Canada. *Wildlife Biol.* **13**, 31–37. (doi:10.2981/0909-6396(2007)13[31:FHOWGG]2.0.CO;2)
78. Powell, R. A. 1981 Martes pennanti. *Mamm. Species* , 1–6.
79. Rosalino, L. M. & Santos-Reis, M. 2009 Fruit consumption by carnivores in Mediterranean Europe. *Mamm. Rev.* **39**, 67–78. (doi:10.1111/j.1365-2907.2008.00134.x)
80. Balestrieri, A., Remonti, L. & Prigioni, C. 2009 Exploitation of food resources by the Eurasian badger (Meles meles) at the altitudinal limit of its alpine range (NW Italy). *Zoolog. Sci.* **26**, 821–827. (doi:10.2108/zsj.26.821)
81. Cleary, G. P., Corner, L. A. L., O'Keeffe, J. & Marples, N. M. 2009 The diet of the badger Meles meles in the Republic of Ireland. *Mamm. Biol.* **74**, 438–447. (doi:10.1016/j.mambio.2009.07.003)
82. Begg, C. M., Begg, K. S., Du Toit, J. T. & Mills, M. G. L. 2003 Sexual and seasonal variation in the diet and foraging behaviour of a sexually dimorphic carnivore, the honey badger (Mellivora capensis). *J. Zool.* **260**, S0952836903003789. (doi:10.1017/S0952836903003789)
83. Begg, C. M., Begg, K. S., Du Toit, J. T. & Mills, M. G. L. 2005 Life-history variables of an atypical mustelid, the honey badger Mellivora capensis. *J. Zool.* **265**, 17–22. (doi:10.1017/S0952836904005990)
84. Chuang, S. A. & Lee, L. L. 1997 Food habits of three carnivore species (Viverricula indica,

Herpestes uwa, and Melogale moschata) in Fushan Forest, northern Taiwan. *J. Zool. London* **243**, 71–79. (doi:10.1111/j.1469-7998.1997.tb05757.x)
85. Zhou, Y. B., Zhang, L., Kaneko, Y., Newman, C. & Wang, X. M. 2008 Frugivory and seed dispersal by a small carnivore, the Chinese ferret-badger, Melogale moschata, in a fragmented subtropical forest of central China. *For. Ecol. Manage.* **255**, 1595–1603. (doi:10.1016/j.foreco.2007.11.018)
86. Gompper, M. E. 1995 Nasua narica. *Mamm. species*. , 1–10. (doi:10.2307/3504195)
87. Valenzuela, D. 1998 Natural history of the white-nosed coati, Nasua narica, in a tropical dry forest of western Mexico. *Rev. Mex. Mastozoología* **3**, 26–44.
88. Gompper, M. E. & Decker, D. M. 1998 Nasua nasua. *Mamm. Species* , 1–9. (doi:10.2307/3504444/2600752)
89. Slater, G. J. 2015 Iterative adaptive radiations of fossil canids show no evidence for diversity-dependent trait evolution. *Proc. Natl. Acad. Sci.* **112**, 4897–4902. (doi:10.1073/pnas.1403666111)
90. Wang, X., Tedford, R. H. & Taylor, B. E. 1999 *Phylogenetic systematics of the Borophaginae (Carnivora, Canidae)*. New York, NY: American Museum of Natural History.
